# Association of neonatal necrotizing enterocolitis with myeloid differentiation-2 and GM2 activator protein genetic polymorphisms

**DOI:** 10.3892/mmr.2015.3499

**Published:** 2015-03-17

**Authors:** WEI ZHOU, WEIMING YUAN, LONGGUANG HUANG, PING WANG, XIAO RONG, JUAN TANG

**Affiliations:** Department of Neonatology, Guangzhou Women and Children’s Medical Center, Guangzhou Medical University, Guangzhou, Guangdong 510120, P.R. China

**Keywords:** myeloid differentiation-2, GM2 activator protein, gene polymorphism, neonatal necrotizing enterocolitis

## Abstract

The aim of the present study was to investigate the association of neonatal necrotizing enterocolitis (NEC) with myeloid differentiation-(MD-2) and GM2 activator protein (GM2A) genetic polymorphisms. Gene resequencing of the MD-2 and GM2A gene exons was performed on 42 neonates, diagnosed with NEC (NEC group), as well as in the rs 11465996 locus, located in the MD-2 gene promoter region. The aim was to detect the genetic polymorphisms present in the neonates with NEC and compare the functional polymorphic loci with 83 neonates without NEC (control group), who had been born during the same period. A polymorphic locus with abnormal frequency was detected in the exon region of the MD-2 gene. In the NEC group, the frequency of genotypes carrying the low frequency allele (G) in the rs 11465996 locus (MD-2 promoter region) was significantly higher compared with the control group (χ^2^=4.388, P=0.036). Furthermore, the frequencies of genotypes carrying the low frequency A and C alleles in the rs1048719 (GM2A gene exon 1) and rs2075783 loci (GM2A intron), respectively, were significantly higher in the NEC group compared with the control group (χ^2^=4.316, P=0.038; and χ^2^=13.717, P=0.000, respectively). In addition, the rs 11465996 polymorphism in the MD-2 gene promoter region was found to be associated with the severity of NEC. Furthermore, the rs2075783 polymorphism in the GM2A gene exon 1 and the rs1048719 polymorphism in the intron region of this gene, were associated with the occurrence of NEC. The present study demonstrated that gene polymorphisms of MD-2 and GM2A were associated with the occurrence or severity of NEC; however, further in-depth exploration is required to clarify the associations between genetic predispositions to polymorphisms, and NEC.

## Introduction

Neonatal necrotizing enterocolitis (NEC) is a severe intestinal disease that occurs predominantly in preterm infants and is one of the leading causes of mortality in neonatal intensive care units (NICUs). According to a previous study, the incidence of NEC in live births was 1–3%, while the rate in preterm infants with a birth weight of ≤1500 g was ~10% (1). Severe cases required surgical treatment, and a mortality rate of ~25% was observed (1). However, preterm birth and low birth-weight do not provide a full explanation on the incidence of NEC, since even with exposure to similar environmental conditions or interference, NEC occurred only in certain newborns (particularly premature infants). In addition, each case exhibited significantly different degrees of disease severity. In certain cases, only minor bloating occurred, while in others, intestinal perforation and necrosis developed rapidly. Therefore, certain neonates may be more likely to develop NEC, possibly due to genetic predisposition.

A previous study demonstrated that excessive activation of the innate intestinal immune response and the inflammatory cascade were involved in the pathogenesis of NEC (2). Myeloid differentiation-2 (MD-2) and GM2 activator protein (GM2A) are members of the MD-2-related lipid-recognition (ML) family. MD-2 is an important component of the intestinal Toll-like receptor 4 (TLR4) innate immune signaling pathway (3). MD-2 is an essential accessory molecule, required for the binding of lipopolysaccharides (LPSs) by TLR4 (4). MD-2 has been demonstrated to bind TLR4 and form a heterodimer, allowing the formation of a complete binding site for LPS (5). Cells expressing TLR4 alone, or TLR4 and mutational MD-2 exhibited a low LPS reactivity (6). Thus, genetic polymorphisms of the MD-2 gene promoter or exon site may significantly affect the transcriptional activities of the MD-2 gene or affect the LPS-induced signal transduction (7), thereby producing an abnormal immune response. GM2A is known to be an inflammatory regulator, with a β-cup structure that is able to bind platelet activating factor (PAF) and hydrolyze it into the inactive form, lyso-PAF (8). As an endogenous phospholipid that regulates inflammation, PAF exhibits important biological activities. PAF-mediated signaling cascades activate nuclear factor-κB, signal transducers and activation of transcription 3 (STAT3), resulting in transcription and immune response, induced by a series of inflammatory factors (9). Thus, PA F is hypothesized to be closely associated with the development of NEC (10). MD-2 and GM2A have been proposed to be associated with the occurrence of NEC (11,12); however, no conclusive evidence exists demonstrating that the expression or structural changes of these factors involved in the pathogenesis of NEC. In the present study, a gene sequencing method was used to resequence the MD-2 and GM2A exons, as well as the rs11465996 locus region (located in the MD-2 gene promoter region) of 42 neonates, which were diagnosed with NEC (NEC group). The aim of the present study was to investigate the presence of genetic polymorphisms in these gene fragments. Subsequently, a comparative analysis was conducted between the functional polymorphic loci of the NEC neonates and 83 neonates without NEC, who were born in the same period (control group), in order to investigate the association between genetic polymorphisms of the MD-2 and GM2A genes and the incidence or severity of NEC.

## Materials and methods

### Clinical data

In total, 42 neonates diagnosed with NEC (Bell’s stage≥II) at the NICU of Guangzhou Women and Children’s Medical Center (Guangzhou, China) between June 2011 and May 2012 were enrolled in the study. These infants were termed as the NEC group, which included 25 full-term (59.5%) and 17 preterm infants (40.5%). In total, 20 infants underwent surgery (47.6%; including 14 full-term and 6 preterm infants), while 22 did not (52.4%). The gestational age range of the NEC group was 28–40 weeks, and the body weight at birth ranged between 1,230 and 3,750 g. The gestational age range of the control group was 31–40 weeks, and the body weight at birth ranged between 1,400 and 3,750 g. Furthermore, 83 neonates without NEC, who had been hospitalized in the NICU during the same period, were enrolled in this study. Follow-up examination at 2 months after birth was used to exclude infants with gastrointestinal inflammation or malformation. Samples from these 83 neonates were used as the control group, which included 48 preterm and 35 full-term infants. Neonates with a history of serious infections and anoxic asphyxia, as well as those with congenital malformations in organs, including the brain, heart, gastrointestinal tract, kidney and respiratory tract, were excluded from this study.

### Specimen collection and processing

Peripheral venous and arterial blood (1 ml) was collected from each infant in the NEC and control groups in an EDTA anticoagulant tube (Yaohua Pharmaceutical Packing Co., Ltd., Laiwu, China), following diagnosis with NEC or birth, respectively. The blood samples were sub-packaged in 1.5 ml Eppendorf tubes (Eppendorf AG, Hamburg, Germany) (250 *μ*l in each tube) and then stored at −80°C for subsequent experiments.

### Extraction of genomic total DNA and amplification of target fragments

A GD2311 Blood gDNA Miniprep kit (Biomiga, San Diego, CA, USA) was used, and total DNA extraction of whole blood samples was performed according to the manufacturer’s instructions. Primer Premier 5.0 software (Premier Biosoft International, Palo Alto, CA, USA) was then used to design the primers and amplify the target fragments. The primers used in the PCR analysis are shown in Table I and were synthesized by Shenzhen BGI Tech Institute (Shenzhen, China).

### Gel electrophoresis and purification of polymerase chain reaction (PCR) products

Using the PCR products, 2% agarose gel electrophoresis (horizontal electrophoresis EPS 300, horizontal electrophoresis tank and Gel Doc XR gel imaging system; Bio-Rad Laboratories, Inc., Hercules, CA, USA) was performed and the results were compared with the marker (DL700 DNA Marker; Takara Bio Inc., Otsu, Japan) in order to verify whether the amplified products had the same sizes as the target fragments. A 96-well purification plate (EMD Millipore, Billerica, MA, USA) was subsequently used to purify the PCR products, according to the manufacturer’s instructions. Briefly, the PCR products were added to the 96-well purification plate alongside 100 *μ*l ddH_2_O, and incubated for 10 min at room temperature. The plate was then dried using a vacuum pump for 10 min. A further 40 *μ*l ddH_2_O was added to the plate, and the plate was incubated at room temperature for 10 min. The purified products were then transferred to another 96-well plate.

### Gene sequencing

The PCR amplification products were sequenced by the Shenzhen BGI Tech Institute using the Sanger method with the ABI 3730XL DNA analyzer (Applied Biosystems, Foster City, CA, USA).

### Statistical analysis

SPSS 16.0 software (SPSS, Inc., Chicago, IL, USA) was used for statistical analysis. Quantitative data are expressed as the mean ± standard deviation and enumerated data are expressed as ratios. Comparisons between pairs of binary data were performed using the χ^2^ test. P<0.05 was considered to indicate a statistically significant difference.

## Results

### Detection of polymorphic loci in the MD-2 exon region

Among the 42 infants with NEC, detection of the functional polymorphic loci was not possible in the MD-2 gene exon region, as exon 3 was smaller, with only 53 bp. However, no studies in the Single Nucleotide Polymorphism database (dbSNP; http://www.ncbi.nlm.nih.gov/SNP/; accessed May, 2012) or the associated literature have reported polymorphisms in this region. As the fragment is small, the SNP database was unable to identify associated functional SNPs.

### Polymorphic loci of the MD-2 promoter region C-1625 G (rs11465996)

**Figure 1:**
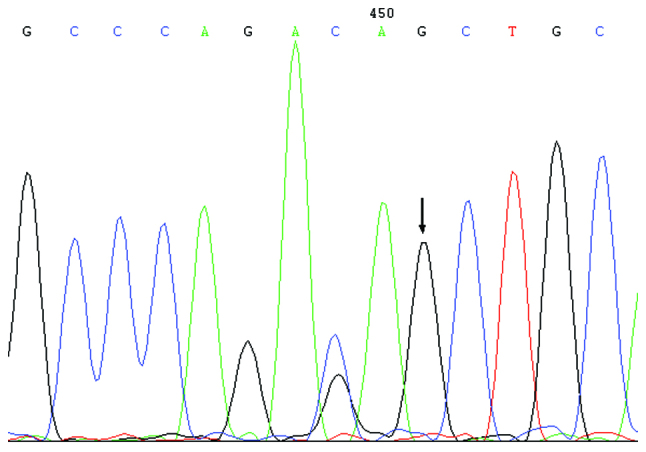
Resequencing peak diagram of MD-2 gene promoter-1625 region, with the arrow-pointed locus being the heterotic locus (single nucleotide polymorphism locus, rs11465996). MD-2, myeloid differentiation-2.

Two genotypes, namely C/C and C/G, were detected in the rs 11465996 locus of the NEC and control groups, where G was the low frequency allele (Fig. 1). The C/G genotype frequencies in the NEC and control groups were found to be 38.1 and 30.1%, respectively, with no statistically significant difference between these groups. In addition, the frequencies of the G allele in the two groups were found to be 19.0 and 15.1%, respectively. The frequencies of the C/G genotype and G allele detected in the present study were lower compared with those previously reported for the Chinese population in the dbSNP (40.0 and 22.2%, respectively) (http://www.ncbi.nlm.nih.gov/snp/). Amongst the neonates with NEC who underwent surgery, the C/G genotype frequency was 55%, which was significantly different compared with the control group (30.1%; P=0.036). Thus, the G allele may be a risk factor for the development of NEC [odds ratio (OR), 1.66; 95% confidence interval (CI), 1.092–3.054]. In the NEC group, the frequency of the C/G genotype was found to be higher in the neonates who had undergone surgery compared with the non-operated neonates, as well as in the full-term infants compared with the preterm infants; however, these differences were not statistically significant (P>0.05; Table II).

### Polymorphisms in the GM2A gene

A total of three missense mutations with high frequencies were detected in the GM2A exon region. Furthermore, a polymorphic locus with abnormal frequency was detected in the intron region. The positions of these loci, the frequency of low-frequency alleles and the frequency of genotypes carrying the low frequency allele in the various groups are shown in Table III.

**Figure 2:**
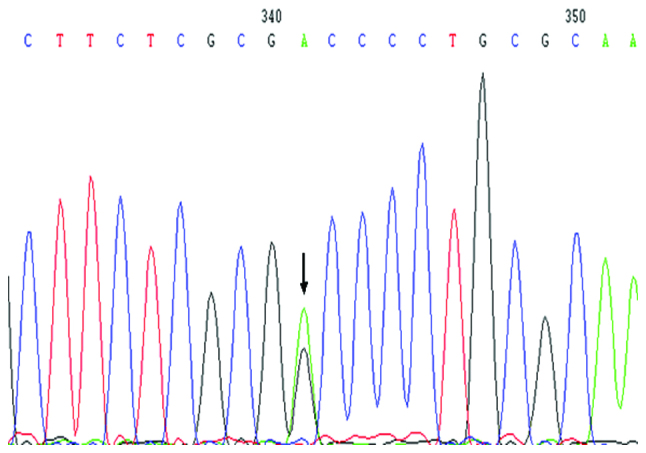
Polymorphic locus of rs1048719 in GM2A gene exon 1 (arrow). This is a heterotic locus, representing the G/A genotype. GM2A, GM2 activator protein.

rs1048719 (G>A) was the single nucleotide polymorphism (SNP) locus of the exon 1 region, where A was the low frequency allele (Fig. 2). In the NEC Group, the frequencies of the A allele and the genotypes carrying the A allele were significantly increased, when compared with the control group (P=0.008 and 0.038, respectively). The A/A and A/G genotypes, which carry the A allele, may be associated with the pathogenesis of NEC (OR, 1.857; 95% CI, 1.037–3.326). In the NEC surgery group and the control group, the frequency of genotypes carrying the A allele (six cases) was higher compared with the control group, although the difference was not statistically significant (P=0.364). However, the A allele frequency of the full-term infants with NEC was significantly higher compared with the control group (χ^2^=5.374, P= 0.020).

rs153477 (A>G) was the SNP locus of exon 2 and possessed a missense mutation. The frequencies of the G allele and genotypes carrying the G allele in this locus exhibited no statistically significant differences among the various groups (in the Chinese population, A was the low frequency allele; (http://www.ncbi.nlm.nih.gov/snp/)). The A allele of the rs1048723 (A>G) locus was a stop codon nucleotide in one of the variants of GM2A. In another variant of GM2A, this locus was located in the coding region. In the present study, the polymorphism of this locus did not exhibit a statistically significant difference among the groups (P>0.05).

**Figure 3:**
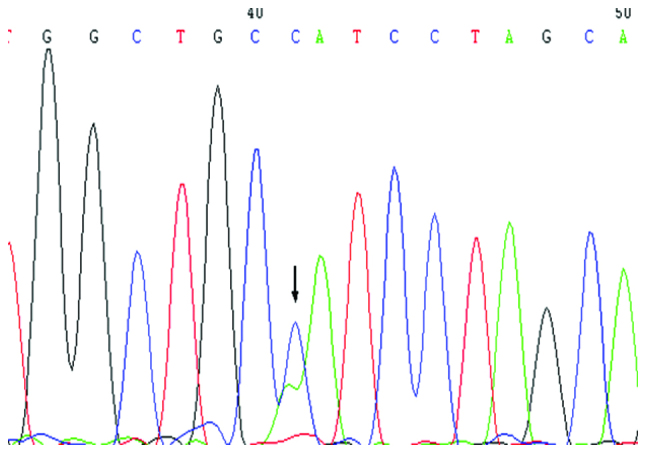
Polymorphic locus of rs2075783 in the GM2A gene intron (arrow). This is a heterotic locus, representing the A/C genotype. GM2A, GM2 activator protein.

**Figure 4:**
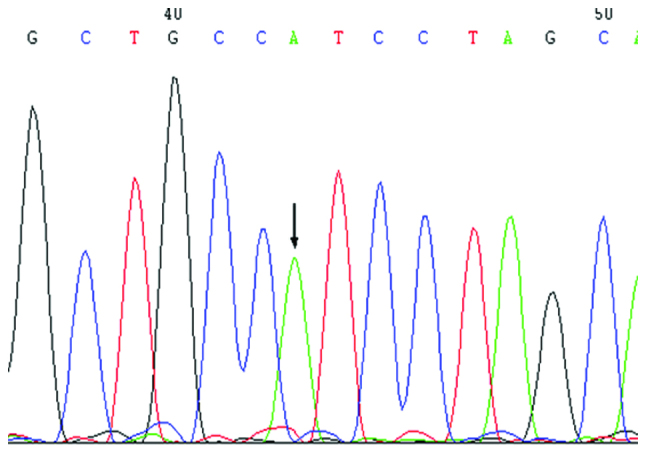
Polymorphic locus of rs2075783 in the GM2A gene intron (arrow). This is a homozygous mutation locus, representing the C/C genotype. GM2A, GM2 activator protein.

Within the intron region that was upstream of exon 4, the rs2075783 (A>C) polymorphic locus was detected, which exhibited statistically significant differences as compared with the NEC group (Figs. 3 and 4). In this locus, C was the low frequency allele. The frequency of genotypes carrying the C allele in the NEC group exhibited a statistically significant difference compared with the control group (χ^2^=13.717, P<0.001). Thus, genotypes carrying the C allele may be a risk factor for the development of NEC (OR, 3.234; 95% CI, 1.685–6.206). Furthermore, the frequency of genotypes carrying the C allele was significantly higher in the NEC surgery and the full-term infants groups compared with the control group (P<0.05).

## Discussion

NEC is a severe gastrointestinal disease that occurs in the neonatal period and an important cause of neonatal morbidity and mortality resulting from disorders of the gastrointestinal tract (13). Although epidemiological studies and other fundamental research have greatly improved the understanding of the disease, the exact cause of NEC remains unclear, while no fully effective methods of prevention or treatment exist currently (14). A further issue, leading to uncertainty in clinical practice, is that NEC also occurs in full-term infants. Although there have been advances in the management of preterm infants, no significant reduction was observed in the incidence of NEC, indicating that neonates developing NEC may possess a genetic predisposition. A number of studies have been conducted, demonstrating the roles of genetic factors in the pathogenesis of NEC. For instance, Bhandari *et al* (15) performed a multicenter retrospective study in twins. The results demonstrated that the genetic background may affect the morbidity associated with NEC. Furthermore, numerous studies have investigated the association between polymorphisms of inflammatory cytokines and NEC (16). The present study investigated the association among polymorphisms in the MD-2 gene and the incidence and severity of NEC.

MD-2 is an important cofactor in the TLR signaling pathway and an important component of the CD14-TLR4/M D - 2 receptor complex, which is able to identify components of the bacterial cell wall (17). Previous studies have demonstrated that the polymorphic locus, rs11465996 (C>G), in the MD-2 promoter region-1625 position significantly affects the efficiency of MD-2 transcription (6,7,18). A mutation in the exon 1 region (amino acid no. 35; Thr35Ala) may weaken the signal that is activated by LPS (19), and the G56R polymorphism may decrease the binding ability of endotoxin (20).

In the present study, no polymorphic loci with abnormal frequency were detected in the MD-2 gene exon region in neonates with NED, indicating that the incidence of NEC may not be associated with structural changes in the MD-2 protein. Hamann *et al* (19) and Vasl *et al* (20) revealed that missense mutations in the MD-2 gene exon region lead to variations in the MD-2 structure, which resulted in a decrease in the binding capacity of the CD14-TLR4/MD-2 receptor complex towards LPS, thereby weakening the LPS-induced signal. However, the mutations reported in the aforementioned studies were very rare and may not be associated with the development of NEC.

The polymorphic locus of rs11465996 (C>G) in the MD-2 gene promoter region-1625 position has also been investigated in previous studies (6,7,18). Changes in allele frequency of this locus may significantly affect the transcription efficiency of the MD-2 gene. Zeng *et al* (6) identified that, in trauma patients, the rs11465996 polymorphic locus was significantly correlated with a higher incidence of sepsis and multiple organ dysfunction (MOD) scores. In addition, Gu *et al* (7) demonstrated that polymorphism of this locus may affect the activity of the MD-2 promoter. In individuals carrying the G allele, LPS-stimulation resulted in a significant increase in the expression levels of whole blood leukocyte MD-2 mRNA and tumor necrosis factor-α. In the Chinese Han population, this polymorphic locus was shown to be associated with MOD and sepsis following severe trauma (18). In the present study, the frequency of the C/G genotype in the NEC and control groups was lower compared with the frequency reported in the dbSNP; however, the difference between these two groups was not statistically significant. However, when compared with the control group, the frequency of this genotype was significantly higher in the severe cases of NEC (neonates who had undergone surgery; P=0.036). This indicated that the G allele of this locus (C/G genotype) is associated with the severity of NEC. In the 42 neonates with NEC, the frequency of the C/G genotype was higher in the surgery group compared with the non-surgery group, and in the full-term infants group compared with the preterm infants group; however, these differences were not statistically significant. These results indicated that the polymorphism of C-1625 G in the MD-2 gene promoter region may be associated with the severity of NEC. Based on the results of the present and previous studies, this may be associated with the polymorphism effect on the transcriptional activities of the MD-2 gene. The abnormal transcription of MD-2 gene may influence the innate immune function of enteric ducts, reducing the effectiveness of the immune regulation of external pathogen stimuli during the process of intestinal flora colonization process and leading to an abnormally amplified inflammatory response. However, the exact underlying mechanism remains unclear and further investigations of the genetics and mechanism are required.

GM2A is another member of the ML family, which binds and inhibits PAF, thereby inhibiting PAF stimulation-induced intracellular calcium release. As an endogenous inflammation-regulatory phospholipid, PAF is known to be important in a variety of pathophysiological processes, including inflammation, allergic reactions, asthma, tumor formation and apoptosis (21). Caplan *et al* (22) demonstrated that the plasma PAF levels of infants with NEC increased, while the level of PAF acetylhydrolase (PAF-AH) was significantly reduced. PAF-AH deficiency has also been observed in preterm infants (23). Soliman *et al* (24) demonstrated that PAF induced the expression of TLR4 in rat intestinal mucosa, as well as the mRNA and protein expression levels of TLR4 in intestinal epithelial cells. In addition, PAF was found to promote the secretion of IL-8 and induce the phosphorylation and nuclear translocation of the epithelial cell STAT3, thus initiating the transcription of pro-inflammatory cytokines.

Previous studies have hypothesized that GM2A may inhibit PAF (25,26). In addition, Rigat *et al* (12) investigated the effects of GM2A in animal models of NEC and demonstrated that the administration of recombinant GM2A inhibited lethal rat intestinal necrotic injury induced by LPS and PAF. The administration of recombinant GM2A was found to significantly reduce the intestinal tissue damage and was also involved in the maintenance of the integrity of the intestinal tight junctions. These functions also implied that GM2A was involved in the prevention of NEC. Whether the interaction of GM2A with PAF is affected as a result of changes to its structure or expression, thereby leading to the development of diseases, including NEC, has not yet been reported.

In the present study, an SNP locus with abnormally increased frequency was identified in the GM2A coding region, namely rs1048719 (G>A), which was located in exon 1. The mutation detected at this locus was a missense mutation, which led to variations in amino acids. Statistical analysis demonstrated that the frequency of the genotypes carrying the low frequency A allele, in the NEC group was significantly higher compared with the control group (P<0.05). Thus, the polymorphism at this locus may be associated with the development of NEC (OR, 1.857; 95% CI, 1.037–3.326), and the A allele may increase susceptibility to this disease. Since the G>A mutation of this locus resulted in variations to the codon (missense mutation), the increase in susceptibility to NEC, induced by the polymorphism at this locus, may be due to changes in the structure of GM2A that decrease the binding and hydrolytic abilities of GM2A to PAF. In addition, an abnormally amplified inflammatory response may be mediated, resulting in the development of NEC. However, this mechanism remains hypothetical, and further investigation is required to establish whether and how a variation to a single base may result in significant changes in the spatial structure of GM2A.

Furthermore, due to the sequencing technology used, 100–200 bp fragments in the upstream and downstream regions of the target fragment were also included when amplifying the fragments of interest. The results revealed that the rs2075783 polymorphism in the intron region exhibited statistically significant differences between the NEC and control groups (P<0.001), and that the frequency of the genotypes carrying the C allele was significantly higher in the full-term NEC group compared with the preterm NEC group (P=0.014). This was a notable result, as this locus is not directly involved in the coding of the GM2A protein. However, whether this polymorphism directly affects the transcription of the GM2A gene or impacts upon the regulation of other genes, thus increasing the NEC susceptibility, is unclear. To the best of our knowledge, no studies have been conducted on the function of this SNP locus. In fact, >95% genes of human genome were found to be functional; however, our understanding of genes continues to exhibit limitations, demonstrating that further research is required.

In conclusion, the present study indicated that the polymorphism, rs11465996, in the MD-2 gene promoter region is associated with the severity of NEC and that the rs1048719 polymorphism in the GM2A gene exon 1 and the rs2075783 polymorphism in the intron region are associated with the occurrence of NEC. However, studies using larger samples are required in order to verify these associations. Furthermore, the development of NEC cannot be explained by a single gene. Further investigation is required into the interactions between various polymorphisms, as well as innate immune and inflammatory factors, and how these are able to singly and synergistically affect the development of NEC.

## Figures and Tables

**Table I:** Primer of each amplified fragment.

Amplification region	Primers (5′→3′)	Annealing temperature (°C)
MD-2-1	(F): AAGAGGAAACAGTTGGATAGGA (R): GAGAAAGATGACGCAGGGA	55
MD-2-2	(F): GCCACATTGCTGATGTCATT (R): TGCTGTGTTAAGCCACAAAGA	55
MD-2-4	(F): AAAGCCTCTGAAATAGTAGCA (R): ACAAACACTCTTGCCCAC	55
MD-2-p^a^	(F): TATCTGGCCCTGTTCTGTCC (R): ATGGTGGCACACACCTGTAA	63.5
GM2A-1	(F): CCGTTCCAGCCGCCTTCA (R): TCTCAGCCAGACCCGCACA	55
GM2A-2	(F): CCAAAGGCCAATTAGGTCAG (R): CTTCACAGTTCCCCAAGCAT	55
GM2A-3	(F): TGGTATGTTTGCCCTGGAAT (R): AGCCGCACAAGATGAGAGAC	55
GM2A-4	(F): ACAGTGCTATGGCCGTCTCT (R): CCCTGGGCTATCAAGAACTG	54

a MD-2-p was the MD-2 gene promoter-1625 locus area. The fragment of MD-2 gene exon 3 was short (53 bp), and no polymorphic loci were detected in the dbSNP database; thus, it was not investigated in the present study. MD-2, myeloid differentiation-2; GM2A, GM2 activator protein; F, forward; R, reverse.

**Table II:** Distributions of the C/G genotype and G allele in each group.

Group	Cases (n)	Genotype (cases, n)		C/G genotype frequency (%)	G allele frequency (%)
		C/C	C/G		
Control	83	58	25	30.1	15.1
NEC	42	26	16	38.1^a^	19.0
NEC surgery	20	9	11	55.0^b^	27.5
NEC non-surgery	22	16	6	27.3^c^	13.6
NEC full-term infants	25	14	11	44.0^d^	22.0
NEC preterm infants	17	12	5	29.4^e^	14.7

Comparison of C/G genotype frequency:

a P=0.370 and χ^2^=0.805, vs. control group;

b P=0.036 and χ^2^=4.388, vs. control group;

c P=0.067 and χ^2^=3.343, vs. NEC non-surgery group;

d P=0.339 and χ^2^=0.913, vs. NEC preterm infants group. NEC, necrotizing enterocolitis.

**Table III:** Low frequency allele frequencies and genotype frequencies that carried the low frequency allele among the groups.

Polymorphic locus	Position	Mutation type	Low frequency allele frequency (%)				Genotype frequencies that carried the low frequency allele (%)			
			NEC group	Control group	Surgery group	Full-term infants group	NEC group	Control group	Surgery group	Full-term infants group
rsl048719	Exon1	m	23.8^a^	10.9^b^	17.5	24.0	38.1^e^	20.5^d^	30.0	40.0
rs153477	Exon2	m	58.8	66.2	63.2	66.7	80.0	88.2	84.2	91.7
rs1048723	Ter	–	23.8	34.3	30.0	32.0	42.9	49.4	50.0	56.0
rs2075783	Intron	–	23.8^e^	10.8^f^	27.5	32.0	42.9^?^	13.3^h^	45.0	56.0

Comparison between a and b, χ^2^=6.981, P=0.008; comparison between c and d, χ^2^=4.316, P=0.038; comparison between e and f, χ^2^=7.275, P=0.007; comparison between g and h, χ^2^=13.717, P=0.000. NEC, necrotizing enterocolitis; m, missense mutation; Ter, stop codon.
